# Tuning Electrical Conductivity and Ultrafast Optical Nonlinearity of Reduced-GO Films Ablated by Femtosecond Laser Direct Writing

**DOI:** 10.3390/molecules30020348

**Published:** 2025-01-16

**Authors:** Youliang Tao, Xuefeng Zhang, Han Wang, Zhongquan Nie, Deng Pan

**Affiliations:** 1 College of Aeronautics and Astronautics, Taiyuan University of Technology, Taiyuan 030024, China; taoyouliang00@163.com; 2 Institute of New Carbon Materials, College of Materials Science and Engineering, Taiyuan University of Technology, Taiyuan 030024, China; 3 Key Lab. of Advanced Transducers and Intelligent Control System, Ministry of Education and Shanxi Province, College of Electronic Information and Optical Engineering, Taiyuan University of Technology, Taiyuan 030024, China; 4 Information Materials and Intelligent Sensing Laboratory of Anhui Province, Anhui University, Hefei 230601, China

**Keywords:** femtosecond laser, reduced graphene oxide films, energy storage devices, electrical conductivity, ultrafast optical nonlinearity

## Abstract

Carbon-based nanomaterials with excellent electrical and optical properties are highly sought after for a plethora of hybrid applications, ranging from advanced sustainable energy storage devices to opto-electronic components. In this contribution, we examine in detail the dependence of electrical conductivity and the ultrafast optical nonlinearity of graphene oxide (GO) films on their degrees of reduction, as well as the link between the two properties. The GO films were first synthesized through the vacuum filtration method and then reduced partially and controllably by way of femtosecond laser direct writing with varying power doses. Subsequently, the four-point probe measurements of the reduced-GO (r-GO) films were demonstrated to exhibit superior resistivity and electrical conductivity compared with the pristine-GO counterpart. It was found that the conductivity of the film increases and then decreases with increasing ablation laser power (*P*), and GO was completely reduced at *P* = 100 mW, with a resistivity and electrical conductivity of 1.09 × 10^−3^ Ω·m and 9.19 × 10^2^ S/m, respectively. GO was over-reduced at *P* = 120 mW, with its resistivity and electrical conductivity being 3.72 × 10^−3^ Ω·m and 2.69 × 10^2^ S/m, respectively. We further tested the ultrafast optical nonlinearity (ONL) of the as-prepared pristine and reduced GO with the femtosecond Z-scan technique. The results show that the behavior of ONL is reversed whenever GO is reduced in a controlled manner. More interestingly, the higher the ablation laser power is, the stronger the optical nonlinearity of r-GO is. In particular, the nonlinear absorption and refraction coefficients of the r-GO films reach up to 3.26 × 10^−8^ m/W and −1.12 × 10^−13^ m^2^/W when *P* = 120 mW. The nonlinear absorption and refraction coefficients reach 1.9 × 10^−8^ m/W and −3 × 10^−13^ m^2^/W, respectively, for *P* = 70 mW. GO/r-GO thin films with tunable photovoltaic response properties have potential for a wide range of applications in microelectronic circuits, energy, and environmental sustainability.

## 1. Introduction

Within the graphene family, graphene oxide (GO) has garnered significant attention in recent years because of its distinctive physical and chemical properties [1]. It serves as an excellent precursor material, thanks to the unique physical and chemical characteristics that arise from the hybridization of sp^3^ and sp^2^ carbon atoms [2]. One of the most intriguing and unique properties of GO is its electrical and optical properties [3], which can be dynamically tuned through the manipulation of the content of oxygen-containing groups by means of physical or chemical reduction [4]. This reduction can be achieved by a variety of methods, including chemical [5], thermal [6], and laser direct writing [7], resulting in precise control of the composition and structure of the material [8,9,10]. In particular, the technique of ultrafast laser direct writing on GO films is highly adaptable and cost-effective [11]. It enables localized reduction and precise control over the patterning of GO films, making it appropriate for a diverse array of applications [12]. The elimination of oxygen-containing functional groups from the surface of GO films via laser direct writing effectively restores the sp^2^ hybridized carbon domains [13,14]. Consequently, the process yields reduced graphene oxide (r-GO), allowing for the adjustment of the electrical and optical characteristics of GO films [15]. This feature endows GO films with a novel advantage in the progression of sophisticated functional nanomaterials for energy storage and environmental remediation, potentially initiating a fresh surge in the advancement of functional nanomaterials [16,17].

The pathway for laser-reduced GO to obtain r-GO with tunable optoelectronic properties has been extensively studied. Zheng et al. analyzed the in situ third-order nonlinear response of GO films during laser reduction, which can be dynamically tuned by varying the laser input dose, and β is deduced to be 40,000 cm/GW at 32 µJ/cm^2^ [18]. V.G. Sreeja et al. reported the effect of reduction time on the third-order optical nonlinearity of r-GO with a nonlinear absorption coefficient of −3.22 × 10^−6^ cm/W for 24 h of reduction [19]. Ma et al. compared the resistance of r-GO at different laser doses, and the lowest resistance of the samples obtained under 10 mW laser power irradiation was about 10 kΩ [10]. Thangavelu et al. reported electrical conductivity as high as 1.73 × 10^−4^ S/m for GO films irradiated for 180 s at a laser energy of 50 mJ [20]. Although many research groups are mainly limited to the dependence of a single property on the level of the reduction process, research on the relationship between the degree of reduction and various properties is limited, and there is a scarcity of studies exploring the interconnections among these different properties in view of the increasingly important role that laser-processed GO is playing in the field of advanced optoelectronic related equipment. Accordingly, it is necessary to investigate the dependence of the conductivity and ultrafast optical nonlinearity of GO films on their reduced degree, as well as the connection between the two properties, to provide a guiding direction for advanced optoelectronic devices.

In this paper, we propose the use of femtosecond laser direct-write reduction in GO to control the oxidation level of GO. The tuning behavior of the resistivity and ONL of r-GO thin films with different laser powers was investigated. The results show that the conductivity of r-GO film is best when the laser energy reaches 100 mW, and the resistivity gradually increases and the conductivity gradually decreases with the increasing laser power. Furthermore, the nonlinear saturated absorption and self-defocusing effects of r-GO films are reversed after femtosecond laser processing compared to pristine GO films. The ONL of r-GO films is gradually enhanced with the gradual increase in laser power. The ONL absorption coefficient of the film processed at 120 mW laser power more than doubles, and the refraction coefficient increases by an order of magnitude compared to the film processed at 70 mW laser power. The results of this paper not only explore the dependence of electrical conductivity and ultrafast optical nonlinearity of GO thin films on their reduced degree, but also explain the connection between the two properties, which provides important insights into r-GO in the field of functional nanomaterials for energy and environmental sustainability.

## 2. Experimental Details

### 2.1. Synthesis of GO Films via Vacuum Filtration Deposition

**Figure 1:**
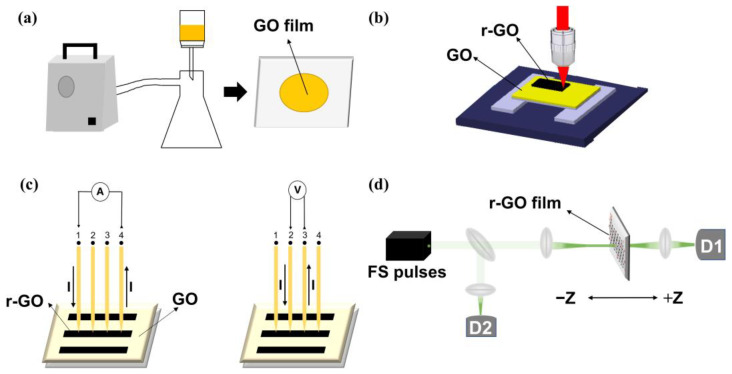
(**a**) Preparation process of GO films. (**b**) Schematic diagram of the detailed process of femtosecond laser direct write ablation of GO. (**c**) Schematic diagram of sheet resistance measurement of r-GO film using a 4-point probe station, and each metal probe (Cu-Au-plated) has a size of 0.74 mm. The arrows represent the direction of the electric current. (**d**) Schematic diagram of measurement of ONL of GO/r-GO film.

The GO films are prepared using the vacuum filtration process (Figure 1a). Briefly, 0.6 mL of GO dispersion (sheet size: 50–200 nm, concentration: 2 mg/mL) was diluted with 50 mL of deionized water. The solution underwent ultrasonication for 40 min (temperature: 25 °C, frequency: 40 Hz) and then slowly flowed into an inverted conical flask, placed on the aluminum filter membrane (filter membrane pore size: 0.02 µm, diameter: 47 mm) with a support ring. The films were formed within 1 h by adjusting the pumping pressure. After filtration, the film was naturally dried for 1 day, and subsequently, the filter membrane was peeled off in water to transfer the obtained graphene oxide film onto a clean glass sheet (13 × 13 mm, thickness: 1 mm). Finally, a GO film sample (~549.5 nm thick) was obtained by natural drying for one day.

### 2.2. Femtosecond Laser Processing

Next, we discuss the removal of oxygen-containing functional groups by direct femtosecond laser ablations of GO films. In this experiment, the mode-locked titanium-sapphire oscillator femtosecond laser (Coherent Inc., Saxonburg, PA, USA), with a central wavelength of 800 nm, a pulse width of 75 femtoseconds and a repetition frequency of 80 MHz, was used for the direct writing of reduced-graphene oxide films. First, the laser processing pattern was designed using AutoCAD 2018. After that, the patterns were loaded onto a femtosecond laser direct-writing device, the relevant laser parameters were set, and the GO films were fabricated using different laser powers (70 mW, 100 mW, 110 mW, and 120 mW) to obtain r-GO with different degrees of reduction (labeled r-GO/70 mW). The detailed process of femtosecond laser processing of GO is shown in Figure 1b.

### 2.3. Material Characterization

The morphology of GO/r-GO films was analyzed using a Japan Electron JSM-7900 model (JEOL, Akishima, Japan) scanning electron microscope (SEM) and a BTM-40 orthogonal metallographic microscope (Batuo, Shanghai, China). The thickness of the GO/r-GO films was characterized using a Dektak XT stylus profilometer (Bruker, Karlsruhe, Germany). The UV-vis absorption spectra of GO/r-GO were analyzed using a Cary 5000 UV-Vis-NIR (Varian Inc., Palo Alto, CA, USA) spectrophotometer. The vibrational states of the elements in the GO/r-GO thin films were characterized using a Thermo Scientific ESCALAB Xi+ (Thermo, Waltham, MA, USA)-type X-ray photoelectron spectrometer (XPS). Raman spectroscopy of GO/r-GO thin films was carried out using a Renishaw Qontor-type (Renishaw, Wotton-under-Edge, UK) focusing Raman spectrometer.

### 2.4. Electrical Conductivity and Ultrafast Optical Nonlinearity Measurement

The sheet resistance of r-GO was measured using the 4-point probe station method (Figure 1c). The resistivity and electrical conductivity are then calculated from the following relationships:

(1) $$\rho =\frac{R_{s}S}{l}$$

(2) $$\sigma =\frac{1}{\rho }$$

In the formula $R_{s}$ is the sheet resistance, l is the electrode spacing (distance between electrodes 2 and 3 in Figure 1c), S is the cross-sectional area, $\rho \;\left(\Omega \cdot m\right)$ is the resistivity, and σ (S/m) is the electrical conductivity. These values were calculated to obtain the electrical conductivity and resistivity of r-GO.

The ONL responses of both the GO and r-GO films were investigated using the fs pulse Z-scan technique (Figure 1d). The light source employed was a 532 nm, an 80 MHz repetition rate, and a 200 fs pulse width ultrafast fiber laser (Chameleon Compact OPO-Vis, Coherent). The tested laser energy density is always less than the processing laser energy density. The laser beam is focused through a convex lens (focal length *f* = 150 mm). The transmittance of the film is measured as a function of the input flux, and the film to be measured is driven by a potentiometric stage moving along the Z-axis (0.67 mm/s) to vary the input flux. The optical power values are recorded by an optical power meter (Newport 2936-R, Irvine, CA, USA).

## 3. Results and Discussion

**Figure 2:**
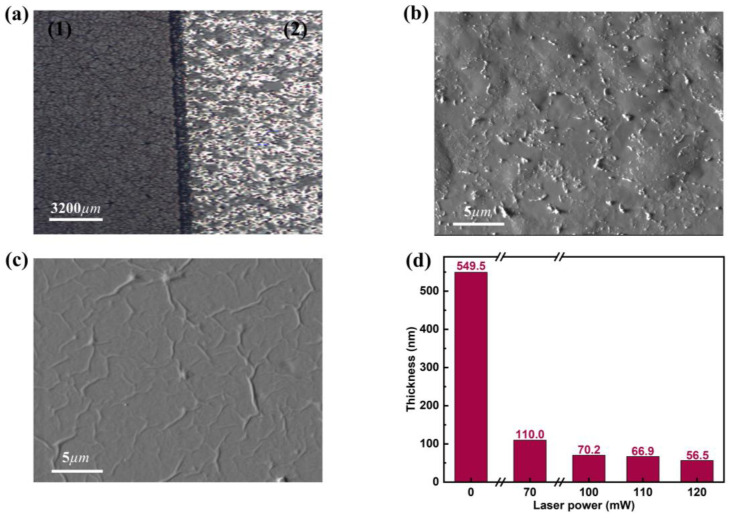
(**a**) GO/r-GO optical microscope images at a scale of 3200 µm, (1) for GO and (2) for r-GO. (**b**) SEM image of GO thin film after femtosecond laser processing with a scale bar of 5 µm. (**c**) SEM image of pristine GO film with a scale bar of 5 µm. (**d**) Stylus profilometer results of GO films fabricated with different laser powers; the thickness of the original GO film is ~549.5 nm.

The surface morphology of GO/r-GO films was characterized by scanning electron microscopy (SEM). From Figure 2c, the surface of the pristine GO film is smooth with some wrinkles, which may be due to uneven pumping during the fabrication of the GO film or some wrinkles during the film transfer process. The surface of r-GO obtained after femtosecond laser processing becomes rough and shows a chipped structure (Figure 2b). The thickness of the GO/r-GO film surface was characterized using a stylus profiler. The results show that the film thickness of the initial GO film is ~549.5 nm and the film thickness for r-GO/70 mW is 110 nm. Figure 2d shows that an increase in laser power leads to an increase in ablation depth and a gradual thinning of the film in the fabricated area.

**Figure 3:**
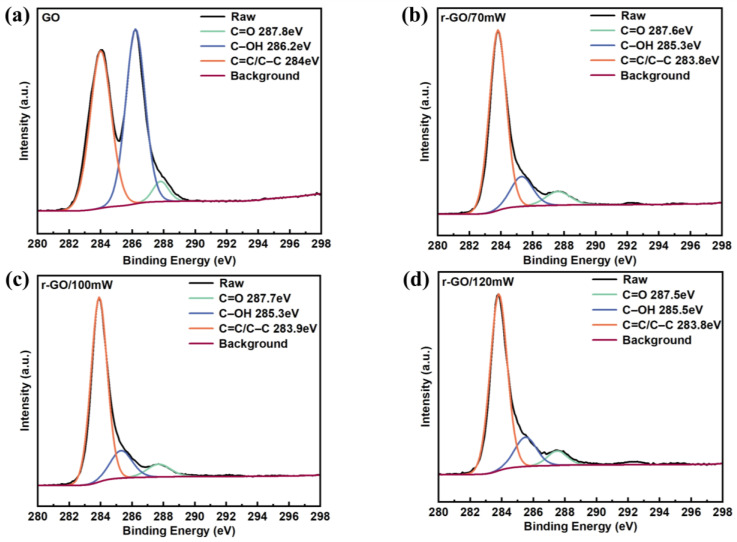
(**a**) The C1s spectrum of GO film. (**b**) The C1s spectrum of r-GO/70 mW. (**c**) The C1s spectrum of r-GO/100 mW. (**d**) The C1s spectrum of r-GO/120 mW.

The chemical element valence states and chemical compositions of GO/r-GO were analyzed by X-ray photoelectron spectroscopy (XPS) [21]. Figure 3b shows that the C1s in the r-GO films exhibit the characteristic configurations of sp^2^ and sp^3^ carbon, single-bonded carbon–oxygen (C–OH), and double-bonded carbon–oxygen (C=O) at 283.8 eV, 285.3 eV, and 287.6 eV, respectively [6,12]. Compared with pristine GO (Figure 3a), r-GO/70 mW (Figure 3b) exhibits reduced signal intensity at 285.3 eV and 287.6 eV, suggesting a reduction of oxygen-containing functional groups after laser action. As shown in Table 1, with the gradual increase in the femtosecond laser power, the content of C=C increases from 47.37% (GO) to 79.07% (r-GO/120 mW), and the content of C–OH decreases from 48.10% (GO) to 13.73% (r-GO/120 mW). The results show that with the increase in laser power, the content of oxygen-containing functional groups gradually decreases, and the degree of reduction in GO films increases gradually.

**Figure 4:**
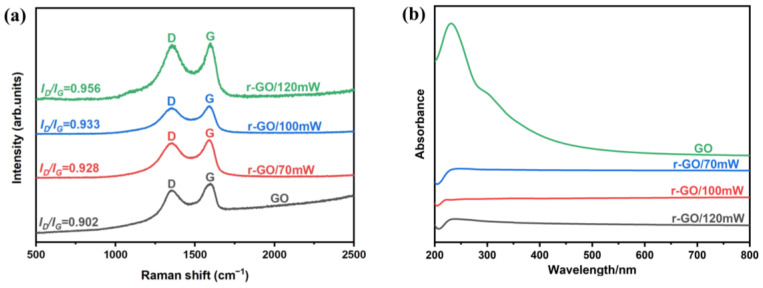
(**a**) Raman spectral characterization results of GO, r-GO/70 mW, r-GO/100 mW, and r-GO/120 mW. (**b**) UV-vis absorption spectra of GO, r-GO/70 mW, r-GO/100 mW, and r-GO/120 mW.

To further evaluate the changes in the structural composition of GO films after laser reduction, we performed Raman spectroscopy on GO/r-GO films. As shown in Figure 4a, both GO and r-GO exhibit peaks D and G. The peak G of pristine GO is located at ~1596 cm^−1^, and the peak G of r-GO/70 mW is located at ~1581 cm^−1^, respectively. R-GO/70 mW is closer to the pristine graphite compared to the pristine GO (peaks D and G of the pure graphite are located at 1340 cm^−1^ and 1566 cm^−1^, respectively), providing further evidence that GO has been reduced, consistent with the XPS analysis. As the laser power increases, the position of peak G of r-GO gradually approaches that of graphite peak G, thus making it more certain that the degree of GO reduction rises with increasing laser power. In addition, the concentration of defects in carbon materials is mainly related to the *I_D_*/*I_G_* strength ratio. As shown in Table 2, the *I_D_*/*I_G_* increases from 0.902 (r-GO/70 mW) to 0.956 (r-GO/120 mW) as the laser power rises from 70 mW to 120 mW, which indicates that more defects are produced in the GO film with the increase in laser power [10].

The UV-vis absorption spectra of GO, r-GO/70 mW, r-GO/100 mW, and r-GO/120 mW are illustrated in Figure 4b. The absorption spectrum of GO displays a strong broad absorption at around 235 nm, attributed to π⟶π∗ transitions of aromatic C=C bonds [17]. By contrast, the absorption of r-GO film is broadband (about 235 nm~445 nm). The absorption of r-GO/70 mW is stronger than that of the r-GO/120 mW film at the same wavelength.

**Figure 5:**
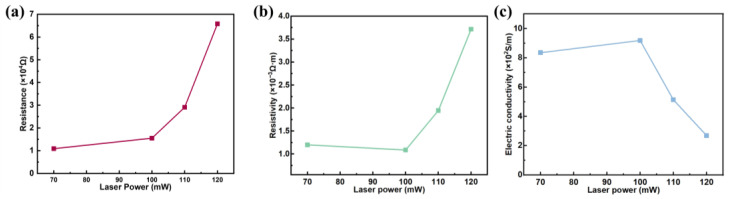
(**a**) Resistance results of r-GO/70 mW, r-GO/100 mW, r-GO/110 mW, and r-GO/120 mW measured by 4-point probe station method. (**b**) Relationship between laser power and resistivity. (**c**) Relationship between laser power and electric conductivity.

Synthesized GO films with very low electrical conductivity are shown in Table 3, where GO electrical conductivity is approximately 6.8 × 10^−8^ S/m [22]. After laser processing to remove the oxygen-containing functional groups on the surface, the carrier density and mobility are enhanced and the electrical properties are improved. To analyze how the conductivity of the r-GO film is controlled by laser power, the thin-layer resistance of r-GO was measured using a four-point probe station (Figure 5a). The resistivity (Figure 5b) and conductivity (Figure 5c) of the r-GO film were then calculated using Equations (1) and (2). Figure 5c shows the relationship between laser power and r-GO electrical conductivity. Overall, the clear trend is that electrical conductivity increases and then decreases with increasing laser power. Specifically, the r-GO/70 mW electrical conductivity increases to 8.35 × 10^2^ S/m compared to the unreduced GO (Table 4). The XPS results confirm that at a laser power of 70 mW, many oxygen-containing functional groups are removed, thereby increasing the electrical conductivity, a state known as the incompletely reduced state. When the laser power is increased to 100 mW, the electrical conductivity reaches a maximum (9.19 × 10^2^ S/m). According to the results of Raman spectroscopy, as the laser power increases more and more, the sp^3^ domain is converted to the sp^2^ domain, and the density and mobility of carriers increase. The conductivity is best at this point, which is the fully reduced state. As the laser power continues to increase, the electrical conductivity gradually decreases and is lowest when the laser power increases to 120 mW (2.69 × 10^2^ S/m). It should be noted that the r-GO film becomes very thin as the laser power continues to increase, as shown in the stylus profilometer measurements in Figure 2d. This causes the carrier density to decrease, which reduces the electrical conductivity of the r-GO film. We call this state over-reduced.

**Figure 6:**
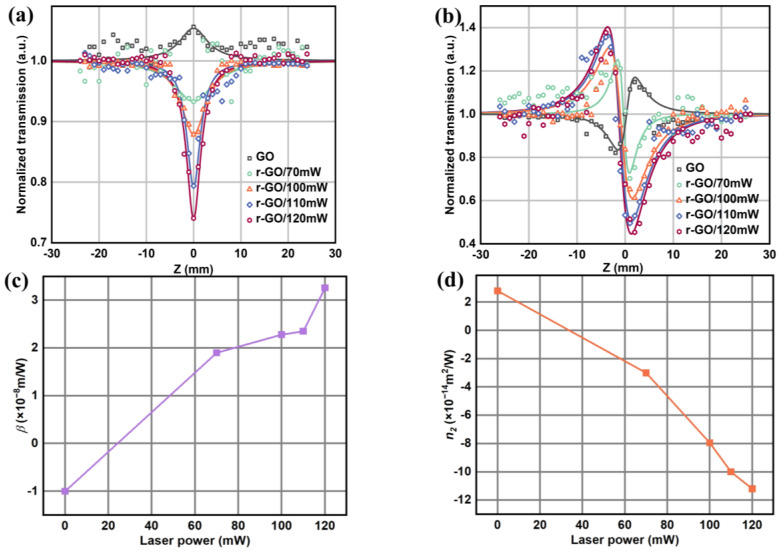
Optical nonlinear measurements of quantitative GO/r-GO films by Z-scan (fs laser) with an energy density of (4.164 × 10^13^ W/m^2^) were obtained for the open-aperture diameter (**a**) and closed-aperture diameter (**b**) Z-scan results. (**c**) Trend of ONL absorption coefficient with laser power. (**d**) ONL trend of refraction coefficient with laser power.

Here, GO and r-GO films were measured using the Z-scan technique to analyze the relationship between laser power and ONL. As shown in Figure 6a,b, the nonlinear saturated absorption and self-defocusing effects are observed in GO films after femtosecond laser processing. The open-aperture Z-scan measurements at the same input flux (Figure 6a) show a peak value of 1.015 for GO and valleys of 0.74, 0.794, 0.879, and 0.935 for r-GO, respectively, indicating a more pronounced nonlinear saturated absorption phenomenon compared to GO films and r-GO films. As shown in Figure 6b, the peak–valley difference is 0.207 for GO and 0.438, 0.533, 0.797, and 0.799 for r-GO for close-aperture Z-scan measurements at the same input flux. The self-defocusing phenomenon for r-GO/120 mW is 1.6 times that of r-GO/70 mW (2.5 times that of self-defocusing). Therefore, it is possible to control the ONL intensity of the r-GO film by adjusting the laser power.

The nonlinear absorption coefficient β can be estimated using the following relationship [25]:

(3) $$\beta =\frac{2\sqrt{2}\cdot △T}{I_{0}\cdot L_{eff}}$$

where (△T) is the open-aperture nonlinear transmittance, where $L_{eff}=-e^{-\alpha L}/\alpha$, L is the thickness of the film, and the laser beam intensity α at $I_{0}=z=0$ is the linear absorption coefficient.

The value of *n*_2_ can be evaluated by the following equation [26]

(4) $$n_{2}=\frac{\lambda △\phi_{0}}{2\pi I_{0}L_{eff}}$$

The peak–valley transmittance difference $△T_{P-V}$ and the nonlinear phase shift $\left|△\phi_{0}\right|$ are in accordance with the following relationship [27]:

(5) $$△T_{P-V}=0.406\left(1-S\right)\left|△\phi_{0}\right|$$

In the formula, $S=1-e^{-2r_{a}^{2}/\omega_{a}^{2}}$ is the linear aperture transmittance, $r_{a}$ is the aperture radius, and $\omega_{a}$ is the laser spot radius before the aperture, while $\left|△\phi_{0}\right|=kn_{2}L_{eff}I_{0}$, k=2π/λ, λ is the wavelength of the laser beam.

The third-order optical nonlinearity ($\chi^{3}$) is a complex variable, which consists of the real part $\left(R_{e}\chi^{3}(esu)\right)$ and the imaginary part $\left(I_{m}\chi^{3}(esu)\right)$. They have the following relation:

(6) $$\left|\chi^{3}\right|={\left[{\left(R_{e}\chi^{3}\left(esu\right)\right)}^{2}+{\left(I_{m}\chi^{3}\left(esu\right)\right)}^{2}\right]}^{\frac{1}{2}}$$

According to the open-aperture Z-scan coefficient β and close-aperture Z-scan to the nonlinear refractive index *n*_2_, the real part $\left(R_{e}\chi^{3}(esu)\right)$ and imaginary part $\left(I_{m}\chi^{3}(esu)\right)$ of the third-order optical nonlinear ($\chi^{3}$) are obtained as follows [28,29]:

(7) $$R_{e}\chi^{3}\left(esu\right)=\frac{\varepsilon_{0}c^{2}n_{0}^{2}}{\pi }n_{2}10^{-4}\left(\frac{cm^{2}}{W}\right)$$

(8) $$I_{m}\chi^{3}\left(esu\right)=\frac{\varepsilon_{0}c^{2}n_{0}^{2}\lambda }{4\pi^{2}}\beta 10^{-2}\left(\frac{cm}{W}\right)$$

where $\varepsilon_{0}$ is the dielectric constant in a vacuum system, *n*_0_ is the linear refractive index, and c is the speed of light in a vacuum.

The third-order optical nonlinearity explains the Kerr nonlinearity effect, which in turn gives rise to effects such as four-wave mixing, self-phase modulation, and cross-phase modulation, and has become the basis for many all-optical signal processing and generation functions [19,30,31]. According to the above relationship, the nonlinear optical parameters, such as nonlinear refractive index, nonlinear absorption coefficient, and the real and imaginary parts of third-order nonlinearity of GO films, reduced by different femtosecond laser powers, are calculated, and Table 5 is obtained.

As shown in Table 5, the ONL of r-GO films is gradually enhanced with the increase in laser power. Before the fully reduced state (r-GO/100 mW), the ONL is gradually enhanced because the C:O ratio in the r-GO film gradually increases with laser power, and the carrier density and mobility are higher. This is consistent with the characterization results of XPS and Raman spectroscopy. The ONL intensity continues to increase with laser power after the fully reduced state, attributed to the fact that the film becomes thin at high power, allowing the ONL intensity to continue to increase (Table 6).

To probe the optical-power-limiting properties of GO and r-GO, the laser pulse energy falling per effective focal spot area is determined from the open-aperture data and is plotted against the normalized transmittance. The position-dependent laser fluence is determined using the following equation [34]:

(9) $$F_{in}\left(z\right)\frac{4\sqrt{ln2}\left(E_{in}\right)}{\left(\pi^{\frac{3}{2}}\right)\omega {\left(z\right)}^{2}}$$

(10) $$\omega \left(z\right)=\omega \left(0\right)\sqrt{1+{\left(\frac{z}{z_{0}}\right)}^{2}}$$

where $F_{in}\left(z\right)$ refers to the laser input fluence at sample position *z*, $\omega \left(z\right)$ represents the beam waist radius corresponding to each *z* position, and $E_{in}$ is the laser input energy.

**Figure 7:**
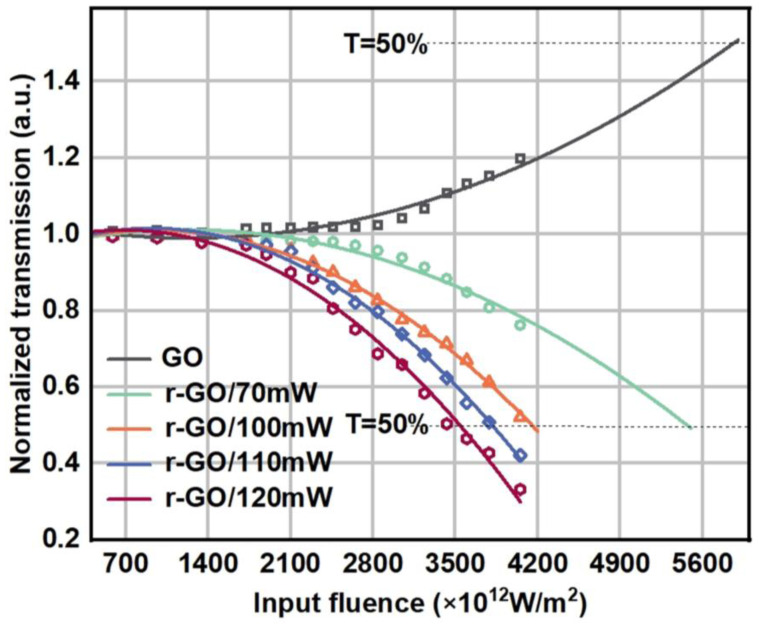
Optical limit of the GO/r-GO under the excitation of linearly and vectorially polarized beams.

Figure 7 shows the optical limiting (OL) curves for GO/r-GO. The results show that the optical limiting efficiency of all r-GO films is significantly higher than that of GO and reversed (the optical limiting threshold is the point at which the transmittance of a material decreases to 50% of its linear value) [35]. The r-GO films have higher sp^2^ domains than GO, and therefore exhibit stronger optical limiting efficiencies [36]. In addition, both GO and r-GO exhibit effective optical power limitation, with the output beam starting to deviate from linearity after reaching a certain threshold (Figure 7) [34]. The starting value of the optical limit for GO dispersion is about 5.85 × 10^15^ W/m^2^, and that of the optical limit for r-GO/120 mW dispersion has the lowest onset value of about 3.5 × 10^15^ W/m^2^, 1.4 times that of GO. These results further demonstrate that the ONL and OL properties of GO are enhanced after femtosecond laser direct writing fabrication. Moreover, the ONL and OL intensities of r-GO rise with the increase in laser power. Chen et al. employed femtosecond laser pulses with a central wavelength of 800 nm, a pulse width of 120 fs, and a repetition frequency of 80 MHz to reduce graphene oxide thin films. The study identified an optimal laser output power range of 3 mW to 8 mW for effective GO patterning [37]. Compared with the pulse width of 100 fs in this paper, the pulse width they used is larger. According to the energy equation $E=P_{peak}\times \tau$, when the energy is certain, the power damage threshold decreases as the pulse width increases. Consequently, they have a lower power threshold. Yung et al. examined the impact of various laser energy densities on the reduction process. The results indicate that as the laser energy increases, a distinct 2D peak emerges, and the degree of reduction progressively enhances [38]. From the above study, we can see that under the conditions of the same power, wavelength and repetition frequency, the pulse width is proportional to the degree of reduction. Energy density is proportional to the degree of reduction. The wavelength is proportional to the damage threshold when other conditions are the same. Electrical conductivity and ONL also increase with the degree of reduction.

The electrical and optical properties of GO/r-GO are closely related to its degree of oxidation. A direct way to describe the degree of oxidation is to assess the ratio of sp^2^ domains; the optical and electrical properties of GO/r-GO are mainly determined by the sp^2^ domain [39]. XPS and Raman spectroscopy results show that more and more sp^3^ domains are converted to sp^2^ domains with increasing laser power. As a result, electrical conductivity and ONL increase with increasing laser power before the r-GO films reach the fully reduced state (r-GO/100 mW), a result attributed to the increase in carrier density and mobility. With further increases in laser power, the high power causes the r-GO films to become very thin. This leads directly to the enhancement of ONL. In addition, the decrease in the carrier density of the thinned film makes the electrical conductivity decrease. The tunability of electrical conductivity and ONL properties are promising solid-state materials for novel optoelectronic devices.

## 4. Conclusions

In summary, we analyze the electrical conductivity and ultrafast optical nonlinearity of GO films with respect to the degree of reduction, as well as the connection between these two properties. The results confirm that the electrical conductivity and ONL of GO films can be modulated by controlling the laser power dosage, and the relationship between the two properties and the laser power dosage is analyzed. The electrical conductivity of GO films increases with the increase in the laser power until it reaches the fully reduced state (r-GO/100 mW). As the laser power continues to increase, the GO films reach the over-reduced state with a subsequent reduction in electrical conductivity. More interestingly, the ONL gradually enhances with increasing laser power. Therefore, we analyze the connection between electrical conductivity and ONL. First, before the laser power reaches 100 mW, the XPS and Raman spectroscopy results confirm that the higher the laser power, the higher the sp^2^ domain content of r-GO, which results in a higher carrier density and mobility. Thus, the electrical conductivity and ONL increase with the laser power before the fully reduced state is reached. The maximum electrical conductivity is 9.19 × 10^2^ S/m (r-GO/100 mW). Secondly, according to the stylus profilometer measurements, the film becomes thin as the laser power continues to increase. Therefore, the electrical conductivity of the r-GO film decreases. This may be due to the decrease in carrier density as the film becomes thinner, making the film less conductive. The ONL continues to be enhanced. The nonlinear absorption coefficients and refraction coefficients of r-GO/120 mW are 1.9 × 10^−8^ m/W, and −3 × 10^−13^ m^2^/W, respectively. This enhanced ONL can be attributed to the fact that a decrease in the thickness of the film causes the material to have enhanced nonlinearities. Graphene oxide thin films with tunable optoelectronic properties and patterning capabilities can be used as a promising new solid-state nanomaterial. In all, GO/r-GO thin films with tunable conductivity and ultrafast optical nonlinear response have a wide range of applications in areas such as energy and environmental sustainability.

## Figures and Tables

**Table 1:** XPS elemental characterization results for GO and r-GO with C=C, C–OH, and C=O functional group occupancies.

Samples	C=C	C–OH	C=O
GO	47.37%	48.10%	4.53%
r-GO/70 mW	75.60%	16.16%	8.24%
r-GO/100 mW	76.75%	15.52%	7.74%
r-GO/120 mW	79.07%	13.73%	7.20%

**Table 2:** Raman spectral characterization results of GO/r-GO, D peak and G peak vertical coordinates, and intensity ratio *I_D_*/*I_G_*.

Samples	D Band (cm^−1^)	G Band (cm^−1^)	*I_D_*/*I_G_*
GO	15,867.2892	17,586.3045	0.902
r-GO/70 mW	11,579.5133	12,472.8161	0.928
r-GO/100 mW	8063.621	8646.4056	0.933
r-GO/120 mW	1722.4261	1801.8036	0.956

**Table 3:** Electric conductivity of relevant materials.

Materials	Electrical Conductivity (S/m)	References
Pristine graphite	15.5	[23]
Reduced graphene nanosheets	10.7	[23]
GO	6.8 × 10^−8^	[22]
Graphene (1.1 mm)	1.3 × 10^2^	[24]

**Table 4:** The r-GO sheet resistance obtained from measurements using a 4-point probe station and the calculated r-GO resistivity and conductivity.

Samples	Thickness (m)	R (Ω)	ρ (Ω·m)	σ (S/m)
r-GO/70 mW	1.10 × 10^−7^	1.09 × 10^4^	1.20 × 10^−3^	8.35 × 10^2^
r-GO/100 mW	7.02 × 10^−8^	1.55 × 10^4^	1.09 × 10^−3^	9.19 × 10^2^
r-GO/110 mW	6.69 × 10^−8^	2.91 × 10^4^	1.95 × 10^−3^	5.14 × 10^2^
r-GO/120 mW	5.65 × 10^−8^	6.58 × 10^4^	3.72 × 10^−3^	2.69 × 10^2^

**Table 5:** Comparison of third-order optical nonlinear coefficients for GO, r-GO/70 mW, r-GO/100 mW, r-GO/110 mW, and r-GO/120 mW films.

Samples	β (m/W)	*n*_2_ (m^2^/W)	$R_{e}\chi^{3}\;(esu)$	$I_{m}\chi^{3}\;(esu)$	$\chi^{\left(3\right)}\;(esu)$	OL (W/m^2^)
GO	−1.00 × 10^−8^	2.8 × 10^−14^	−6.94 × 10^−10^	−8.16 × 10^−10^	1.07 × 10^−9^	5.85 × 10^−15^
r-GO/70 mW	1.90 × 10^−8^	−3.00 × 10^−14^	−4.97 × 10^−11^	1.33 × 10^−10^	1.42 × 10^−10^	5.48 × 10^−15^
r-GO/100 mW	2.28 × 10^−8^	−7.95 × 10^−14^	−3.87 × 10^−10^	4.70 × 10^−10^	6.09 × 10^−10^	4.08 × 10^−15^
r-GO/110 mW	2.35 × 10^−8^	−1.00 × 10^−13^	−1.99 × 10^−9^	1.98 × 10^−9^	2.80 × 10^−9^	3.7 × 10^−15^
r-GO/120 mW	3.26 × 10^−8^	−1.12 × 10^−13^	−4.332 × 10^−9^	5.32 × 10^−9^	6.85 × 10^−9^	3.5 × 10^−15^

**Table 6:** Nonlinear refractive index and nonlinear absorption coefficient of GO and r-GO materials.

Materials	β (m/W)	*n*_2_ (m^2^/W)	References
GO	−14 × 10^−7^	7 × 10^−13^	[18]
r-GO	−2.45~−3.22 × 10^−11^	2.02~2.16 × 10^−12^	[19]
r-GO	5.6 × 10^−13^	7.4 × 10^−17^	[32]
r-GO	3.8 × 10^−11^	−1.4 × 10^−12^	[18]
N-GO	9~10 × 10^−10^	3.9~8.2 × 10^−16^	[33]

## Data Availability

Data is contained within the article.
